# THE CANNABIS AND OLDER PERSONS STUDY: MOVING ALONG A ROAD FROM ANIMAL MODELS TO STATE CAPITOLS

**DOI:** 10.1093/geroni/igad104.0033

**Published:** 2023-12-21

**Authors:** Brian Kaskie

## Abstract

The Cannabis and Older Persons Study has examined the increasing use of cannabis among Americans over 60 years old since 2016. This year’s symposium presents varied methodological approaches researchers have used to better understand the relationship between age and cannabis use. Barry Setlow examines how cannabis use corresponds with age-associated synaptic dysfunction, neuroinflammation, and tau pathology in animal models (younger v older rats), and how such association may contribute to cognitive aging. Nicole Ennis examines the association between cannabis use and simulated driving performance among adults aged 50 and older focusing on response time, attention, and executive functioning tasks that predict on-road performance. Yan Wang examines short and long-term effects of medical marijuana use among older adults using ecological momentary assessments. Divya Bhagianadh analyzes responses provided by 1398 older persons who completed a special module concerning cannabis use as part of the Health and Retirement Survey and applies a multi-level framework to better understand how cannabis use is shaped by individual attitudes and health status. Fadi Martinos considers how state medical cannabis program eligibility criteria and provider qualifications contribute to cannabis access and use among older adults. Together, these studies reflect how scientific understanding of cannabis use and aging has been informed by the application of multiple disciplinary perspectives, and how researchers use different methodological approaches to identify both desirable and undesirable outcomes associated with cannabis use among older adults. This symposium offers policy makers, program administrators and clinicians empirically-based insights concerning how cannabis use affects older adults.

